# Clinical Chemistry

**Published:** 1884-01

**Authors:** 


**Clinical Chemistry.** An account of the analyses of blood, urine, morbid products, etc., with an explanation of some of the clinical changes that occur in the body in disease. By CHAS. HENRY RALFE, M. A., M. D., Contab., Fellow of the Royal College of Physicians, London. Formerly Demonstrator of Physiological Chemistry in the Medical School of St. George's Hospital. Illustrated with sixteen engravings. H. C. Lea's Son & Co., Philadelphia, Pa.

This is another of the excellent series of manuals for students of medicine published by the Leas. Like the others, it is an eminently practical work, furnishing students and practitioners with a concise account of the best methods of examining, chemically, abnormal blood, urine, morbid products, etc., at the bedside or in the laboratory. The value of such knowledge as is here given, to the student and practitioner, is daily becoming better recognized. It ought to be an important feature in medical education.

